# Wnt/β-catenin pathway as a potential prognostic and predictive marker in patients with advanced ovarian cancer

**DOI:** 10.1186/1757-2215-7-16

**Published:** 2014-02-06

**Authors:** Lubomir Bodnar, Aleksandra Stanczak, Szczepan Cierniak, Marta Smoter, Marzena Cichowicz, Wojciech Kozlowski, Cezary Szczylik, Maciej Wieczorek, Monika Lamparska-Przybysz

**Affiliations:** 1 Department of Oncology, Military Institute of Medicine in Warsaw, 128 Szaserów Street, 04-141 Warsaw, Poland; 2 Innovative Drugs Research & Development Department, Celon Pharma Inc, Lomianki, Poland; 3 Department of Pathology, Military Institute of Medicine in Warsaw, 128 Szaserów Street, 04-141 Warsaw, Poland

**Keywords:** β-catenin, E-cadherin, WNT-1, Advanced ovarian cancer, Prognostic marker, Predictive marker

## Abstract

**Background:**

β-catenin is the key protein in the WNT signalling pathway and it forms adherent junctions together with E-cadherin. In ovarian carcinoma, abnormal expression of β-catenin, E-cadherin and WNT-1 was observed, but their prognostic and predictive role is unclear. The aim of this study was to clarify the prognostic and predictive role of E-cadherin, β-catenin and WNT-1 in advanced epithelial ovarian carcinoma (AEOC).

**Methods:**

The expression of E-cadherin, β-catenin and WNT-1 was determined by immunohistochemistry in AEOC. The correlation between expression of these proteins and progression-free survival (PFS) and overall survival (OS) was evaluated. Statistical analyses included Kaplan-Meier estimation, log-rank test, Spearman correlation and Cox proportional-hazards model.

**Results:**

In ovarian cancer, intense expression of E-cadherin, β-catenin and WNT-1 was found. In multivariate analysis, strong membrane β-catenin expression was an independent unfavourable predictor for PFS (HR 2.19, 95% CI 1.09-4.39; p = 0.028), while in univariate analysis, strong membrane β-catenin expression was a prognostic factor for OS in patients with AOC (p = 0.039). In multivariate analysis, only resistance to first-line chemotherapy was an adverse independent prognostic factor for OS (HR 16.84; 95% CI 5.07-55.98; p < 0.0001). Additionally, strong membranous β-catenin expression was associated with resistance to platinum-based chemotherapy (p = 0.027).

**Conclusions:**

These findings support that WNT/β-catenin pathway and E-cadherin are important factors in advanced epithelial ovarian cancer.

## Background

Epithelial ovarian cancer (EOC) is the most common ovarian malignancy and accounts for 90% of cases of ovarian tumours [1]. In 2008, it is estimated that worldwide there were 224,747 women diagnosed with ovarian cancer and 140,163 deaths caused by ovarian cancer [2]. Ovarian carcinoma is the eighth most common cancer and the seventh most frequent cause of cancer death in women [2]. 63% of EOC patients have widespread disease at presentation [3]. Despite surgery and platinum-based systemic treatment the 5-year survival for these patients is poor and accounts for 27% [3]. Therefore, prognostic and predictive factors for better management of EOC patients are required.

The most important prognostic factors for EOC include: age, performance status, histology type, International Federation of Gynaecology and Obstetrics (FIGO) stage and tumour grade [4,5]. However, biochemical and molecular markers are becoming important variables. The most significant marker is the level of cancer antigen 125 (CA-125), which is elevated in approximately 80% of advanced ovarian cancer cases [6]. Serum CA-125 level and its half-life are known to be correlated with OS and PFS [7-9]. Additionally, it is also a marker of response to chemotherapy [7,9-11].

Recently, more attention has been focused on the molecular markers of the WNT/β-catenin pathway, which is widely studied in EOC [12-14]. The WNT/β-catenin pathway is triggered by WNT ligands, while β-catenin is the “heart” of the pathway and activates expression of many important proteins responsible for cell cycle, proliferation and survival e.g. cyclin D1, c-Myc. Moreover, β-catenin, together with E-cadherin, forms adherent junctions mediating cell adhesion [15]. In the absence of WNT ligands, β-catenin binds E-cadherin and forms complexes in the cell membrane. Free cytosolic β-catenin is recruited to a degradation complex constituted by anaphase promoting complex (APC) protein, Axin, glycogen synthase kinase 3β (GSK3β) and casein kinase I (CKI). GSK3β and CKI phosphorylate β-catenin, which is subsequently ubiquitinated by ubiquitin ligase protein (βTrCP) and degraded in the proteasome. WNT-1 binds to the frizzled/lipoprotein receptor-related protein 5/6 (FZD/LRP5/6) receptors and triggers inactivation of the degradation complex. Unphosphorylated β-catenin is then transported to the nucleus, where it binds T-cell factor/lymphoid enhancer factor (TCF/LEF) and activates gene expression of proteins responsible for cell cycle, proliferation and survival [16].

Deregulation of WNT/β-catenin pathway or altered expression of E-cadherin was found in many cancers as well as in EOC [17,18]. One of the possible mechanisms of WNT/β-catenin pathway alteration involves mutations of the β-catenin gene (CTNNB1), which are found in endometrioid subtype of ovarian cancer [19-21]. Additionally, aberrant expression of β-catenin, E-cadherin and WNT-1 was observed in ovarian carcinoma [22-27]. The prognostic role of β-catenin and E-cadherin are disputed, while their impact on response to chemotherapy has never been evaluated in ovarian cancer patients.

The aim of this study was to determine the expression of β-catenin, E-cadherin and WNT-1 in advanced epithelial ovarian cancers and to assess the correlation of expression of the studied proteins with patient survival and response to platinum-based chemotherapy. We found changes in the expression of the studied proteins in ovarian cancer cells. Moreover, strong membrane β-catenin expression was identified as an unfavourable predictor for PFS and was associated with resistance to platinum-based chemotherapy for EOC patients.

## Methods

### Patients

We analysed medical records of all consecutive EOC patients treated in the Department of Oncology at the Military Institute of Medicine in Warsaw, Poland between March 2001 and December 2007. The inclusion criteria were as follows: (1) histologically confirmed advanced epithelial ovarian cancer in FIGO stage III-IV; (2) history of debulking surgery followed by first-line chemotherapy regimen: paclitaxel (135 mg/m^2^) with cisplatin (75 mg/m^2^) or paclitaxel (175 mg/m^2^) with carboplatin (AUC6), administered every 3 weeks for 6 cycles; (3) accessibility of primary tumour specimens and full medical data. Response to first-line chemotherapy according to RECIST criteria (version 1.0), PFS and OS were obtained from medical records and analysed retrospectively. This study was approved by the institutional review board of the Military Institute of Medicine in Warsaw (46/WIM/2009).

### Immunohistochemistry

Immunohistochemical staining was performed on formalin-fixed, paraffin-embedded primary tumours. Tissues were sectioned at 3 μm and were mounted on Super Frost Ultra Plus® slides (Menzel GmbH&co KG) and subjected to antigen retrieval in Target Retrieval Solution, pH 9 (DAKO) with PT Link (DAKO). Tissues were incubated with mouse monoclonal anti-β-catenin antibody (dilution 1:100, clone β-Catenin-1, DAKO), mouse monoclonal anti-E-cadherin antibody (dilution 1:100, clone NCH-38, DAKO) or rabbit polyclonal anti-WNT-1 antibody (dilution 1:100, Spring Bioscience). Negative controls were incubated with mouse or rabbit IgGs (DAKO). Subsequently, sections were incubated with peroxidase-based EnVision™ + system (DAKO). Colorectal epithelium was used as an external positive control showing strong membranous expression of E-cadherin and β-catenin.

### Evaluation of the staining

The expression was scored by three independent observers (AS, SC, WK) without knowledge of the clinical data. All membranous, cytoplasmic and nuclear staining was evaluated in cancer cells. The intensity of membrane staining was categorized as follows: strongly positive when intensity was equal to the intensity of cell membrane in positive control and weakly positive corresponding to the intensities between strong and negative. The presence of cytoplasmic and nuclear staining was graded into two groups: negative and positive if 10% of tumour cells showed immunoreactivity.

### Statistical analysis

Statistical analyses included descriptive statistics with determination of minimal and maximal values, means and medians, with 95% confidence interval (CI) for particular variables. OS was defined as time elapsed between the date of diagnosis and date of death or the date of last follow-up. PFS was defined as the time from diagnosis until disease recurrence or death or date of last follow-up. A Spearman test for non-parametric variables was used to assess correlation between histoclinical data and the expression of studied proteins. A Mann–Whitney U test for non-parametric variables was used to assess if the expression of studied proteins had any predictive value to response to chemotherapy according to RECIST criteria (version 1.0). Univariate analyses of variables influencing PFS or OS were performed by log-rank test, which identified a preliminary list of significant factors. All variables found to be significant and factors that showed a trend towards significance (p < 0.1) in the univariate analysis were included in the multivariate analysis. Multivariate analyses of PFS and OS were performed by Cox proportional-hazards regression using the forward stepwise method. Median and life tables were computed using the product-limit estimate by the Kaplan and Meier method and the log-rank test was employed to assess the statistical significance, p values less than 0.05 were considered as significant. Statistical calculation was performed using the STATISTICA for Windows Version 7.0 software.

## Results

Among 132 patients with epithelial ovarian cancer in our database, 46 were eligible. The main reasons for the exclusion of patients from the study were lack of access to tumour samples (71 patients) and early stage of EOC (15 patients). Patient characteristics are summarized in Table 1. Median age in the study group was 54 years (95% CI; 52.1-57.4). The majority (69.6%) of patients were in stage III of the disease. More than half of the patients had the serous type of ovarian cancer and poorly differentiated tumours (52.2% and 54.4%, respectively). Twenty-seven (58.7%) patients underwent optimal debulking surgery (together with optional interval debulking surgery) and 67.4% of the group was sensitive to first-line chemotherapy.

**Table 1:** Characteristics of patients (n = 46)

**Characteristics**	**n (%)**
Age	
Median 95% CI (years)	54 (52.1-57.4)
Performance status (ECOG scale)	
0	6 (13.1%)
1	37 (80.4%)
2	3 (6.5%)
FIGO stage at diagnosis	
IIIA	4 (8.7%)
IIIB	8 (17.4%)
IIIC	20 (43.5%)
IV	14 (30.4%)
Histologic cell type	
Serous	24 (52.2%)
Endometrioid	10 (21.7%)
Mucinous	3 (6.5%)
Clear cell	2 (4.3%)
Mixed	6 (13.1%)
Undifferentiated	1 (2.2%)
Grade	
G1 and G2	21 (45.6%)
G3 and unknown	25 (54.4%)
Primary surgery (with interval surgery)	
Optimal debulking	27 (58.7%)
Suboptimal debulking	19 (41.3%)
Platinum sensitivity	
Sensitive (>6 months)	31 (67.4%)
Resistant (<6 months)	15 (32.6%)

**Figure 1:**
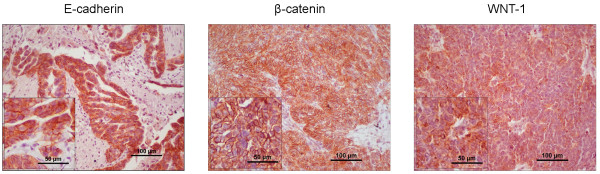
**Expression of E-cadherin, β-catenin and WNT-1 in ovarian cancer.** E-cadherin displayed strong membranous and cytoplasmic immunoreactivity in cancer cells Strong membranous, moderate cytoplasmic and lack of nuclear expression of β-catenin was observed in tumour tissue. In ovarian carcinoma, there was strong WNT-1 immunoreactivity.

In ovarian cancer cells, membrane E-cadherin expression was strongly positive in 39 (84.8%) patients. Moreover the presence of cytoplasmic E-cadherin was observed in tumours of almost all patients (45/46; 97.8%). Expression of β-catenin was strong in almost half of all patients (21/46; 45.6%). Moreover, β-catenin was present in the cytoplasm of EOC cells in 19 patients (41.3%), while it was absent in the cell nuclei in all EOC tumours. In ovarian tumour cells, WNT-1 expression was present in 31 (67.4%) women (Figure 1, Table 1).

**Figure 2:**
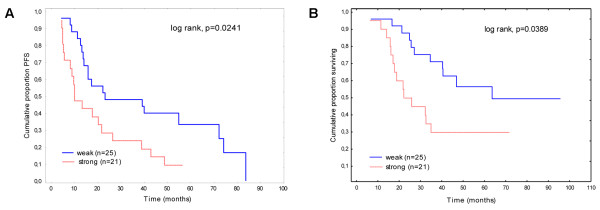
**Kaplan-Meier curves for PFS (A) and OS (B) by expression of membranous β-catenin.** Red line: strong expression of β-catenin. Blue line: weak expression of β-catenin.

To evaluate the impact of analysed proteins and histoclinical variables on EOC patients’ outcome, univariate analysis was performed. The analysis revealed the identification of several prognostic factors for PFS such as histopathologic cell type and membranous β-catenin expression. Age and residual tumour size revealed a prognostic trend for PFS (Table 2). Serous type of tumour was associated with shortened PFS (13.8 mo vs. 23.2 mo; p = 0.028). Younger age and greater residual tumour size showed a trend towards significance, while there was no association between PFS and performance status or tumour grade. Among analysed proteins, only membrane β-catenin was a prognostic factor in univariate analysis. Patients with tumours displaying strong expression of membranous β-catenin had shorter PFS than patients with a decreased level of the studied protein (9.9 mo vs. 22.8 mo; p = 0.024) (Figure 2). The expression of E-cadherin or WNT-1 were not associated with PFS. In the multivariate analysis, only intense expression of membrane β-catenin was an independent adverse prognostic factor for PFS (HR 2.19, 95% CI 1.09-4.39; p = 0.028).

**Table 2:** Univariate and multivariate analysis of progression-free survival

**Clinical parameter**	**Univariate analysis**			**Multivariate analysis**	
	**n (% )**	**Median (months)**	**P value**	**HR (95% CI)**	**P value**
Age					
< 65	38 (82, 6%)	14, 1	0, 076	NS	NS
≥ 65	8 (17, 4%)	23, 2			
Histopathologic cell type					
Serous	24 (52, 2%)	**13, 8**	**0, 028**	NS	NS
Others	22 (47, 8%)	**23, 2**			
Residual tumor size					
<1 cm	19 (41, 3%)	21, 8	0, 054	NS	NS
> 1 cm	27 (58, 7%)	10, 7			
Performance status (ECOG)					
0-1	43 (93, 5%)	16, 7	0, 564		
2	3 (6, 5%)	22, 0			
Tumor grade					
G1, G2	21 (45, 6%)	12, 9	0, 981		
G3, unknown	25 (54, 4%)	17, 5			
E-cadherin membranous					
Negative	7 (15, 2%)	16, 7	0, 775		
Positive	39 (84, 8%)	15, 9			
E-cadherin cytoplasmic					
Negative	1 (2, 2%)				
Positive	45 (97, 8%)	-	-		
β-catenin membranous					
Normal	21 (45, 6%)	**9, 9**	**0, 024**	**2, 19 (1, 09-4, 39)**	**0, 028**
Decreased	25 (54, 4%)	**22, 8**			
β-catenin cytoplasmic					
Negative	27 (58, 7%)	15, 4	0, 806		
Positive	19 (41, 3%)	16, 7			
β-catenin nuclear					
Negative	46 (100, 0%)	-	-		
Positive	0 (0, 0%)				
WNT-1					
Normal	31 (67, 4%)	13, 5	0, 898		
Decreased	15 (32, 6%)	18, 8			

NS - non significant.

In our study, we also found prognostic factors for OS (Table 3). Patients resistant to first-line chemotherapy had shortened OS in comparison to platinum-sensitive tumours (21.7 mo vs. 93.4 mo; p < 0.001). Additionally, serous ovarian carcinoma was associated with shortened OS in comparison to other subtypes (62.5 mo vs. 80.8 mo; p = 0.043). Among analysed proteins, only strong expression of membrane β-catenin was a prognostic factor for shortened OS in univariate analysis (22.2 mo vs. 62.2 mo; p = 0.039; Figure 2). Residual tumour size showed a trend towards significance, while there was no association between OS and age, performance status or tumour grade. In the multivariate analysis, only resistance to first-line chemotherapy was an unfavourable prognostic factor (HR 16.84; 95% CI 5.07-55.98; p < 0.0001).

**Table 3:** Univariate and multivariate analysis of overall survival

**Clinical parameter**	**Univariate analysis**			**Multivariate analysis**	
	**n (% )**	**Median (months)**^**#**^	**P value**	**HR (95% CI)**	**P value**
Age			0, 248		
< 65	38 (82, 6%)	70, 1%			
≥ 65	8 (17, 4%)	75, 0%			
Histopathologic cell type			0, 043*	NS	NS
Serous	24 (52, 2%)	**62, 5%**			
Others	22 (47, 8%)	**80, 8%**			
Residual tumor size			0, 134		
<1 cm	19 (41, 3%)	81, 1%			
> 1 cm	27 (58, 7%)	55, 9%			
Performance status (ECOG)			0, 276		
0-1	43 (93, 5%)	71, 2%			
2	3 (6, 5%)	66, 6%			
Tumor grade			0, 498		
G1, G2	21 (45, 6%)	32, 6			
G3, unknown	25 (54, 4%)	40, 4			
Sensitivity to first-line chemotherapy			<0, 0001*	**16, 84 (5, 07-55, 98)**	<0, 0001*
Resistant (<6 months)	26 (35, 1%)	**21, 7%**			
Sensitive (>6 months)	48 (64, 9%)	**93, 4%**			
E-cadherin membranous			0, 472		
Negative	7 (15, 2%)	50, 0			
Positive	39 (84, 8%)	35, 7			
E-cadherin cytoplasmic	1 (2, 2%)	-	-		
Negative	45 (97, 8%)				
Positive					
β-catenin membranous			0, 039*	NS	NS
Normal	21 (45, 6%)	22, 2			
Decreased	25 (54, 4%)	62, 2			
β-catenin cytoplasmic			0, 916		
Negative	27 (58, 7%)	32, 6			
Positive	19 (41, 3%)	40, 1			
β-catenin nuclear		-	-		
Negative	46 (100, 0%)				
Positive	0 (0, 0%)				
WNT-1			0, 846		
Normal	31 (67, 4%)	33, 7			
Decreased	15 (32, 6%)	40, 5			

^#^If median was not achieved, the results were described as a percentage of patients with 2-year OS; NS- non significant; *The value of the probability of a statistically significant (p <0.05); CI-confidence interval; HR- hazard ratio; ECOG- The Eastern Cooperative Oncology Group scale of *performance status*.

We analysed the association between the intense expression of membrane β-catenin and the response to chemotherapy in the 29 patients with measurable disease according to RECIST criteria (version 1.0). There was a difference observed in complete response (CR), partial response (PR), stable disease (SD) and progressive disease (PD) distribution according to membrane β-catenin expression (p = 0.044). Eight (61.5%) EOC patients with tumours demonstrating strong β-catenin expression in the cell membrane had SD or PD, while most of the patients (14/16; 87.5%) with tumours displaying weak membrane β-catenin expression had an objective response (CR + PR) that was statistically significant (p = 0.027; Table 4).

**Table 4:** Association between membrane β-catenin expression and response to chemotherapy in patients according to RECIST criteria (version 1.0)

**Response to to chemotherapy according to to RECIST**	**Normal membrane β-catenin expression**		**Decreased membrane β-catenin expression**		**Mann–Whitney U test**
	**(n=13)**		**(n=16)**		
	**n**	**%**	**n**	**%**	**P value**
OR (CR+PR)	5	38.5	14	87.5	0.027*
SD+PD	8	61.5	2	12.5	
CR	5	38.5	13	81.25	0.044*
PR	0	0.0	1	6.25	
SD	6	46.1	1	6.25	
PD	2	15.4	1	6.25	

Abbreviations: *The value of the probability of a statistically significant (p <0.05); RECIST *-* Response Evaluation Criteria In Solid Tumours; CR - Complete Response; PR - Partial Response; SD - Stable Disease; PD - Progressive Disease.

## Discussion

In the present study, we analysed the expression of E-cadherin, β-catenin and WNT-1 in advanced ovarian carcinoma. We demonstrated the presence of E-cadherin expression both in the cell membrane and cytoplasm of ovarian tumour cells. The expression of E-cadherin has been investigated in ovarian carcinoma, but in most of the studies, patients in different stages of the disease were examined [24,28]. According to the published data, in the majority of cases of ovarian cancer, E-cadherin is upregulated unlike other type of cancers, where E-cadherin expression is decreased [18]. Ovarian surface epithelium displays epithelial and mesenchymal characteristics and does not contain E-cadherin, but rather expresses N-cadherin [22,29-32].

In primary and well-differentiated cancers E-cadherin expression is high, while in advanced EOC, E-cadherin expression is moderate, although complete loss of E-cadherin is rare [22,23,25,28-30,33]. Moreover, E-cadherin was found in small cohesive tumour nodules floating in the peritoneal cavity, in metastatic lesions and effusion specimens [34-36]. We suggest that in metastatic cancer cells, E-cadherin expression is required for cell survival. There are several studies documenting that E-cadherin contributes to activation of the PI3K/AKT signalling pathway, which controls cellular survival and proliferation [37-39]. In ovarian cancer cell lines, E-cadherin allows the recruitment of PI3K-p85 regulatory subunit to the cell membrane, leading to the activation of the p110 catalytic subunit following signal transduction [39]. Additionally, E-cadherin could also activate the MAPK pathway through RAF [38]. These data indicate that E-cadherin may be important for ovarian cancer cell survival.

Furthermore, we detected intense expression of membrane β-catenin in advanced EOC. Our results are concordant with earlier reports showing positive β-catenin expression in ovarian tumours, but they differ from the results reported by other groups where the expression of β-catenin was decreased [23,25]. Additionally, Davidson et al. found that although there was no difference in the intensity of β-catenin expression, there was a reduction in the number of cells expressing membranous β-catenin [33]. These results suggest that expression of β-catenin is maintained in advanced ovarian carcinomas, but at a moderate level. In vitro studies showed that shRNA-mediated silencing of β-catenin resulted in inhibition of proliferation and decreased capability of colony formation in A2780 ovarian cancer cells [40]. On the basis of these studies, one can speculate that β-catenin is required for proliferation of ovarian cancer cells. A similar effect was observed in colorectal cancer, lung cancer and glioblastoma cells [41-43]. It means that the presence of β-catenin might be required for proliferation and migration, but the precise mechanism is still unknown and should be determined.

β-catenin is also a key component of the signalling pathway that is activated by WNT ligands. We showed that in most of the ovarian tumours, there was a strong WNT-1 expression; however, no nuclear β-catenin was found. It might suggest that the WNT/β-catenin signalling pathway was not activated despite the presence of WNT-1 ligand. WNT-1 expression has been detected in ovarian carcinomas [26,44]; however, other WNT ligands were also found such as WNT-5a, which was highly expressed in EOC tumours [26,45]. Yoshioka et al. studied expression of all WNT ligands at the mRNA level in ovarian tumours. They found that WNT-3 and WNT-4 expression was reduced, while expression of WNT-7a and WNT-7b was increased [46]. Additionally WNT-7a increased expression was confirmed at the protein level [46]. In humans, the WNT protein family consists of 19 ligands that can play a different role in signal transduction. For example, WNT-1 and WNT-2 activates the canonical WNT pathway, where β-catenin is a key protein, whereas WNT-5a, WNT-7a and WNT-7b activate non-canonical pathways including the planar cell polarity pathway or WNT/Ca2+ pathway [47-49]. In ovarian cancers, both types of ligand were found, which implicates that canonical and non-canonical WNT pathways could control ovarian carcinogenesis. Thus, further analysis of all WNT ligands on a larger group of EOC patients is needed.

In our study, we found prognostic factors for OS and PFS of EOC patients and predictors for chemotherapy response. We revealed that strong membrane β-catenin expression was an independent adverse prognostic factor for PFS, while only resistance to first-line chemotherapy was an unfavourable prognostic factor for OS of advanced ovarian cancer patients. Davidson et al. also investigated the influence of β-catenin expression on advanced EOC patient survival, but they did not find any statistically significant correlation [33]. However, low membrane expression of β-catenin was shown to be an adverse prognostic factor for OS of patients in different stages of the disease [27,28]. On the other hand, preserved expression of membrane β-catenin was associated with 10-year disease-related survival and favourable recurrence-free survival of EOC patients in univariate analysis [25]. Discordance between these results and ours could be due to differences between the pattern of gene expression in the advanced ovarian cancer and tumours confined to the primary site. Shirdar et al. investigated genetic differences between stages I/II and III/IV of ovarian tumours [50,51]. They found that in advanced ovarian cancers, there were more chromosomal gains and gene amplifications compared to early carcinomas. Additionally, it has been reported that activation and overexpression of BTAK/Aurora-A, which is essential for chromosome segregation and centrosome function, was associated with early stage EOC, while activation of phosphorylated AKT or SRC was associated with advanced-stage disease [52-54]. The variety of genetic features in early and advanced ovarian cancers may result in a complex array of prognostic factors.

Finally, we found that strong membranous β-catenin expression was associated with the lack of response to chemotherapy, which corroborates in vitro studies. Silencing of β-catenin leads to the increased sensitivity of A2780 ovarian cancer cells to cisplatin, paclitaxel and vincristine [40]. Moreover, metastatic melanoma cells with β-catenin knocked down are more sensitive to cisplatin, temozolomide and doxorubicin [55]. In addition, cisplatin-resistant laryngeal carcinoma cells have increased expression of β-catenin in the cell membrane [56]. It suggests that expression of β-catenin might be associated with the response to chemotherapy. Due to β-catenin being observed in the cell membrane, but not in the cell nuclei, we speculate that β-catenin present in adherent junctions could be important in drug response. One of the known mechanisms of drug resistance is the cell-adhesion-mediated drug resistance (CAM-DR). The association between cell adhesion and resistance of tumour cells to anticancer agents was observed for the first time by Sutherland [57]. Further studies showed correlation between the expression of cell adhesion molecules and drug resistance: collagen VI, collagen XIA1 and connexin 43 were upregulated in cisplatin-resistant ovarian carcinoma cells [58,59], while high expression of claudin-7 was associated with a poor response to platinum-based chemotherapy in EOC patients [60]. Additionally, 25% of overexpressed proteins identified in the carboplatin- and paclitaxel-resistant tissues are the components of the extracellular matrix (i.e. γ-catenin, δ-catenin) [61]. A range of proteins involved in cellular adhesion, including β-catenin, may become new predictors of response to platinum-based chemotherapy in ovarian carcinoma, but more evidence showing their usefulness is needed.

## Conclusions

In conclusion, our study showed the presence of β-catenin and E-cadherin expression in advanced ovarian cancers. Our results imply that β-catenin and E-cadherin expression may be required for ovarian carcinogenesis because these proteins are involved in signalling pathways that control cell proliferation. Additionally, β-catenin could be responsible for resistance to chemotherapy, because some cell adhesion proteins are associated with resistance of tumour cells to chemotherapy. Moreover, we speculate that canonical and non-canonical WNT pathways could control ovarian carcinogenesis, because β-catenin is absent in cancer cell nuclei, despite a strong WNT-1 expression. These findings support that WNT/β-catenin pathway, as well E-cadherin, are important in advanced epithelial ovarian cancer.

## Abbreviations

APC: Anaphase promoting complex; βTrCP: Ubiquitin ligase protein; CA-125: Cancer antigen 125; CAM-DR: Cell-adhesion-mediated drug resistance; CI: Confidence interval; CKI: Casein kinase I; CR: Complete response; EOC: Epithelial ovarian cancer; FIGO: International Federation of Gynaecology and Obstetrics; FZD/LRP: Frizzled/lipoprotein receptor-related protein; GSK3β: Glycogen synthase kinase 3β; OS: Overall survival; PD: Progressive disease; PFS: Progression-free survival; PR: Partial response; SD: Stable disease; TCF/LEF: T-cell factor/lymphoid enhancer factor.

## Competing interests

All authors declare that they have no competing interests.

## Authors’ contributions

LB participated in acquisition of data, performed the statistical analysis and wrote the manuscript. AS carried out immunohistochemical analyses and evaluation of the staining, and wrote the manuscript. SC carried out evaluation of the staining. MS participated in acquisition of data. MC carried out immunohistochemical analyses. WK carried out evaluation of the staining. CS conceived of the study and participated in its design and coordination. MW conceived of the study and participated in its design and coordination and helped to draft the manuscript. MLP conceived of the study and participated in its design and coordination and helped to draft the manuscript. All authors read and approved the final manuscript.

## Acknowledgement

This work was supported in part by a grant from Military Institute of Medicine in Warsaw and by Celon Pharma Inc.
